# Clinicopathological and circulating cell‐free DNA profile in myositis associated with anti‐mitochondrial antibody

**DOI:** 10.1002/acn3.51901

**Published:** 2023-09-18

**Authors:** Yikang Wang, Yawen Zhao, Meng Yu, Luhua Wei, Wei Zhang, Zhaoxia Wang, Yun Yuan

**Affiliations:** ^1^ Department of Neurology Peking University First Hospital Beijing 100034 China; ^2^ Beijing Key Laboratory of Neurovascular Disease Discovery Beijing 100034 China; ^3^ Key Laboratory for Neuroscience, National Health Commission of the People's Republic of China Peking University Beijing 100083 China

## Abstract

**Objective:**

Anti‐mitochondrial antibodies (AMAs) are associated with idiopathic inflammatory myopathies (IIMs). We aimed to summarize the clinicopathological characteristics, assess circulating cell‐free mitochondrial DNA (ccf‐mtDNA), and circulating cell‐free nuclear DNA (ccf‐nDNA) in AMA‐associated IIMs.

**Methods:**

Medical records of 37 IIMs patients with AMAs were reviewed. Circulating cell‐free mtDNA and ccf‐nDNA levels in sera from IIMs patients with AMAs (*n* = 21), disease controls (*n* = 66) and healthy controls (HCs) (*n* = 23) were measured and compared. Twenty‐eight immune‐mediated necrotizing myopathy (IMNM) patients, 23 dermatomyositis (DM) patients, and 15 anti‐synthetase syndrome (ASS) patients were enrolled as disease controls. Correlations between variables were analyzed.

**Results:**

Limb weakness was observed in 75.7% and neck weakness in 56.8% of patients. Cardiac involvement occurred in 51.4% of patients. Muscle pathology revealed 81.1% of IMNM, 5.4% polymyositis, and 13.5% nonspecific myositis. Microinfarction was observed in 8.1% of patients. Serum ccf‐mtDNA levels in AMA‐associated IIMs were significantly higher than those in HCs (*p* < 0.001), but no significant differences between AMA‐associated IIMs and IMNM, DM, or ASS. Serum ccf‐nDNA levels in AMA‐associated IIMs were significantly higher than those in HCs (*p* = 0.02), and significantly lower than those in DM (*p* = 0.02). Serum ccf‐nDNA levels correlated negatively with MMT8 total scores (*r*s = −0.458, *p* = 0.037) and positively with mRS scores (*r*s = 0.486, *p* = 0.025). Serum ccf‐nDNA levels were significantly higher in the non‐remission group (*p* < 0.01).

**Interpretation:**

AMA‐associated IIMs exhibit distinct clinicopathological features. Serum ccf‐nDNA may serve as a potential marker for disease severity and prognosis in AMA‐associated IIMs.

## Introduction

Idiopathic inflammatory myopathies (IIMs) are a group of rare, acquired autoimmune diseases involving skeletal muscles, lung, heart, joints, skin, and other organs or tissue. Myositis‐specific antibodies (MSAs) are commonly correlated with distinct clinical phenotypes and pathological features of IIMs, aiding in accurate diagnosis and potential mechanism insights.^1^, ^2^, ^3^, ^4^ Additionally, myositis‐associated antibodies (MAAs), although not specific to IIMs, can indicate particular phenotypes, whether occurring in isolation or alongside other autoimmune disorders. Anti‐mitochondrial antibodies (AMAs), the hallmark of primary biliary cirrhosis (PBC), have been identified in patients with IIMs since 1973.^5^ Numerous studies have established a correlation between IIMs and AMAs, with some researchers classifying the latter as MAAs.^6^, ^7^ However, a comprehensive understanding of IIMs with AMAs is currently lacking, and discrepancies exist in terms of susceptible age, selective heart involvement, and distribution of muscle weakness.^6^, ^8^, ^9^

Circulating cell‐free DNA (ccf‐DNA), a recently recognized damage‐associated molecular pattern, can trigger an inflammatory response.^10^, ^11^ Circulating cell‐free DNA primarily originates from deceased cells of the hematopoietic lineage, with minimal contributions from other tissues.^12^, ^13^ Mitochondria‐derived ccf‐DNA (ccf‐mtDNA) serves as an indicator of mitochondrial dysfunction,^14^, ^15^, ^16^ and increased levels of nuclear‐derived ccf‐DNA (ccf‐nDNA) have been observed in various disease states, often serving as a marker for disease activity monitoring.^17^, ^18^, ^19^ Elevated ccf‐DNA levels have been detected in the sera/plasma of patients with several conditions, including cancer and autoimmune diseases, notably IIMs.^20^, ^21^, ^22^, ^23^ Anti‐mitochondrial antibodies (AMAs) specifically target immunodominant epitopes containing lipoic acid, particularly the pyruvate dehydrogenase complex situated within the inner mitochondrial membrane,^24^, ^25^ playing complex roles in mitochondrial biological functions.^26^ The potential mitochondrial dysfunction and its correlation with disease phenotype and muscle pathology in IIMs patients with AMAs remain poorly understood. Therefore, in this study, we retrospectively reviewed 37 IIMs patients with AMAs at our center, summarizing their clinical features and morphology. Furthermore, we assessed and evaluated the clinical significance of ccf‐mtDNA and ccf‐nDNA in these patients. Our findings contribute to a better understanding of the disease spectrum and pathogenesis in IIMs with AMAs.

## Methods

### Patients

A total of 37 patients who visited the Department of Neurology at Peking University First Hospital from September 2015 to April 2023 were included in this study. All 37 patients were diagnosed with IIMs with AMAs, following the diagnostic criteria established by the European Neuromuscular Centre.^27^ Diagnostic criteria: (1) subacute or insidious onset; (2) subjective or objective symmetrical weakness of proximal muscles; (3) rash typical of dermatomyositis (DM); (4) elevated serum creatine kinase (CK) level; (5) typical electromyographic findings; (6) typical pathological changes in muscle biopsy.

### Data collection

Demographic and clinical data were collected retrospectively from patients' medical records. Muscle strength was assessed using the Manual Muscle Testing‐8 (MMT8) scale, as proposed by the International Myositis Outcome Assessment Collaborative Study. Cardiac involvement was defined as the presence of cardiac symptoms accompanied by abnormal findings on electrocardiography, echocardiography, or cardiac magnetic resonance imaging. The disease activity of IIMs patients was evaluated using the Myositis Disease Activity Assessment Tool (MDAAT) prior to muscle biopsy. Functional status was assessed using the modified Rankin Scale (mRS). Serum CK levels were recorded from patients prior to muscle biopsy.

### Serum myositis antibody test

Sera samples from patients were stored at −80 C. All patients underwent testing for the following MSAs and MAAs using Euroimmun line tests (Euroimmun, Lübeck, Germany): anti‐transcriptional intermediary factor 1‐γ, anti‐melanoma differentiation‐associated gene 5, anti‐Mi2, anti‐nuclear matrix protein 2 (NXP2), anti‐small ubiquitin‐like modifier activating enzyme 1, anti‐small ubiquitin‐like modifier activating enzyme 2, anti‐signal recognition particle (SRP), anti‐3‐hydroxy‐3‐methylglutaryl‐coA reductase (HMGCR), anti‐Jo‐1, anti‐threonyl‐tRNA synthetase, anti‐alanyl‐tRNA synthetase, anti‐glycyl‐tRNA synthetase, anti‐isoleucyl‐tRNA synthetase, anti‐asparaginyl‐tRNA synthetase, anti‐phenylalanyl‐tRNA synthetase, and anti‐tyrosyl‐tRNA synthetase antibodies (W. H. Zhang, et al. 2022). AMAs were assessed using indirect immunofluorescence, following the manufacturer's protocol (Euroimmun, Lubeck, Germany).

### Quantification of ccf‐mtDNA and ccf‐nDNA levels

Sera from 21 IIMs patients with AMAs, 66 disease controls, and 23 healthy controls were available for measurement of ccf‐mtDNA and ccf‐nDNA levels. Twenty‐eight age‐ and gender‐matched patients with immune‐mediated necrotizing myopathy (IMNM), 23 DM patients, 15 patients with anti‐synthetase syndrome (ASS) were enrolled as disease controls. Among patients with IMNM, 22 patients were positive for anti‐SRP antibodies, 6 patients were positive for anti‐HMGCR antibodies. All disease controls were negative for AMAs. The demographic characteristics of disease controls and healthy controls was presented in Table S1. Serum samples were processed promptly after blood withdrawal, undergoing repeated centrifugation to prevent cell lysis and eliminate potential cell contaminants. To extract ccf‐mtDNA and ccf‐nDNA, a Serum‐Free DNA Extraction Kit with magnetic beads (Tiangen, DP709) was employed. Quantitative real‐time polymerase chain reaction (RT‐qPCR) was conducted following the methodology described in our prior study^15^ to measure the levels of ccf‐mtDNA using the mitochondrial NADH dehydrogenase 1 (*MT‐ND1*) gene and ccf‐nDNA using the beta‐actin (*ACTB*) gene. The reaction mixture, consisting of 20 μL, included 2 μL of circulating cell‐free DNA from serum samples, 1× UltraSYBR Mixture (CW2601), 0.2 μM MT‐ND1 primers or ACTB primers, and nuclease‐free water. The reactions were transferred to sealed 96‐well plates for amplification. Standard cycling conditions were employed for PCR reactions: 95°C for 10 min, followed by 40 cycles of 95°C for 15 sec, and 1 min at 60°C, and a final step of 30 sec at 95°C, 30 sec at 65°C, and 30 sec at 95°C. Agilent Aria software version 1.71 was utilized for the analysis of the qPCR data. ACTB served as the internal reference gene. The quantification of mtDNA copy number was determined according to a previous report,^28^ wherein the mitochondrial‐encoded MT‐ND1 gene and nuclear‐encoded ACTB gene were amplified using serial dilutions of cloned vectors, enabling the generation of a linear standard curve for accurate quantification. The primer sequences are outlined below:

1. MT‐ND1 forward: 5′‐CCACCTCTAGCCTAGCCGTTTA‐3′.
2. MT‐ND1 reverse: 5′‐GGGTCATGATGGCAGGAGTAAT‐3′.
3. ACTB forward: 5′‐CTCCATCCTGGCCTCGCTGT ‐3′.
4. ACTB reverse: 5′‐GCTGTCACCTTCACCGTTCC‐3′.

### Muscle pathology

After obtaining consent forms, open muscle biopsies were conducted on all patients. The biopsies were obtained from either the biceps brachii or quadriceps femoris muscles. Serial frozen sections were stained with hematoxylin and eosin, modified Gomori trichrome, periodic acid‐Schiff, and oil red O and costained for the detection of adenosine triphosphate enzyme (pH 4.5 and 10.8), nicotinamide adenine dinucleotide tetrazolium reductase, nonspecific esterase, succinate dehydrogenase, cytochrome c oxidase‐succinate dehydrogenase, and acid phosphatase. Immunohistochemical staining was carried out on the sections using primary antibodies specific to human CD3, CD4, CD8, CD20, CD68, major histocompatibility complex class I (MHC‐I), membrane attack complex (MAC), as well as dystrophin‐N, C, R, dysferlin, desmin, and α, β, γ‐sarcoglycan.

Two experienced reviewers (WZ and YY), who were knowledgeable in the interpretation of muscle biopsies and muscle immunoanalysis, independently assessed the muscle biopsies. The reviewers were blinded to the underlying myositis antibodies of patients. The muscle pathological patterns were categorized based on the 119th European Neuromuscular Centre criteria^27^ into nonspecific myositis, IMNM, DM, and polymyositis (PM).

Each specimen was evaluated by the reviewers using an optical microscope at 20 × magnification for cell counting. The reviewers, blinded to the specimen type and identity, selected five random areas on the same cryostat section for analysis. In each chosen field, the number of necrotic myofibers, sarcolemmal MAC deposition, and total myofibers were counted. Subsequently, the mean values of the proportion of necrotic myofibers and sarcolemmal MAC deposition were calculated.

### Follow‐up study

The duration of the follow‐up period was determined from the initial visit to the hospital until either the patient's demise or their final examination/visit. Patients who could be reached were encouraged to participate in clinical interviews, while telephone interviews were conducted for those unable to physically visit the hospital. Clinical interviews encompassed inquiries regarding physical signs, clinical symptoms, auxiliary examination findings, and medication outcomes. Telephone interviews involved comprehensive questioning pertaining to constitutional, cutaneous, skeletal, gastrointestinal, pulmonary, and cardiac symptoms, as well as a detailed summary of medications. The disease activities were assessed using the physician's global assessment, which was rated on a 10‐cm visual analog scale (VAS) with scores ranging from 0 to 10. Higher scores indicated more severe disease activity. The assessment of disease activity for each individual was conducted by the same physician. Disease remission was defined as a VAS score of ≤1.

### Standard protocol approvals and patient consent

The study was conducted in accordance with the principles of the Declaration of Helsinki. This study and related protocols were approved by the Ethics Committee at Peking University First Hospital and written informed consent has been obtained from the subjects (or their legally authorized representative).

### Statistical analysis

Categorical data, such as sex, presence of symptoms, and presence of histopathological features, are presented as frequencies and percentages. Quantitative data, including age at disease onset, disease duration, serum CK levels, MMT8 total scores, ccf‐mtDNA, and ccf‐nDNA levels, are reported as median (interquartile range) (IQR). In contrast, MDAAT scores are presented as mean ± SD.

Normality of distribution and homogeneity of variance were assessed using the Shapiro–Wilk normality test and homogeneity of variance test, respectively. Comparisons among the five groups of ccf‐mtDNA, ccf‐nDNA levels, and the ratio of mitochondrial to nuclear DNA were conducted using the Kruskal–Wallis followed by Dunn post hoc test. Comparisons between the two groups were conducted using the Mann–Whitney *U*‐test. The relationship between variables was evaluated using Spearman's correlation tests. A two‐tailed *p* value <0.05 was considered statistically significant.

Statistical analyses were carried out using SPSS version 25.0. Figures were generated using GraphPad Prism 9 and Adobe Photoshop.

## Results

### General characteristics

Our cohort of IIMs patients with AMAs consisted of 30 females and 7 males (Table 1). The median age at disease onset was 51 years (IQR 46–57), and the median disease duration was 12 months (IQR 3–37). The most common initial symptom was limb weakness, reported by 37.8% of patients, followed by cardiac symptoms (32.4%), myalgia (8.1%), and asymptomatic hyperCKemia (13.5%). Arthralgia was the initial symptom in 5.4% of patients, while respiratory failure was present in 2.7% of patients initially.

**Table 1:** Clinical characteristics of IIMs patients with AMAs.

Characteristics	IIMs patients with AMAs (*n* = 37)
Female, *n* (%)	30 (81.1%)
Age at disease onset, median (IQR), years	51 (46, 57)
Disease duration, median (IQR), months	12 (3, 37)
Muscle involvement	
Skeletal muscle involvement	
Limb weakness, *n* (%)	28 (75.7%)
Proximal weakness, *n* (%)	28 (75.7%)
Upper limbs, *n* (%)	17 (45.9%)
Lower limbs, *n* (%)	27 (73.0%)
Neck weakness, *n* (%)	21 (56.8%)
MMT8 total scores, median (IQR)	74.5 (66, 78)
Dyspnea, *n* (%)	14 (37.8%)
Type II respiratory failure, *n* (%)	4 (10.8%)
Dysphagia, *n* (%)	7 (18.9%)
Myalgia, *n* (%)	10 (27.0%)
Muscle atrophy, *n* (%)	2 (5.4%)
Cardiac involvement, *n* (%)	19 (51.4%)
Extramuscular involvement	
Skin rashes, *n* (%)	6 (16.2%)
Arthralgia, *n* (%)	6 (16.2%)
Interstitial lung disease, *n* (%)	10/23 (43.5%)
Weight loss, *n* (%)	12 (32.4%)
Concomitant other autoimmune diseases, *n* (%)	16 (43.2%)
MDAAT score, mean ± SD	6.78 ± 3.359
Serum CK, median (IQR), IU/L	1500 (889, 2655)
MSAs, *n* (%)	7/29 (24.1%)

AMAs, anti‐mitochondrial antibodies; CK, creatine kinase; IIMs, idiopathic inflammatory myopathies; MDAAT, Myositis Disease Activity Assessment Tool; MMT8, Manual Muscle Testing‐8; MSAs, myositis‐specific antibodies. IQR, interquartile range; SD, standard deviation.

Weight loss was observed in 12 (32.4%) patients. Skin rashes were observed in 6 (16.2%) patients. Five (16.2%) patients presented with arthralgia. ILD was observed in 10 out of 23 (43.5%) patients. Additionally, 16 (43.2%) patients had concomitant other autoimmune diseases, including Sjogren's syndrome (three cases), PBC (nine cases), localized scleroderma (one case), glomerulonephritis (one case), rheumatoid arthritis (two case), and autoimmune thyroid disorders (three cases). One (2.7%) patient suffered from scoliosis. The mean MDAAT score was 6.78 ± 3.359. The median serum CK level was 1500 (IQR 889–2655) U/L. MSAs were detected in 7 out of 29 (24.1%) patients. Specifically, 5 out of 29 (17.2%) patients had anti‐SRP antibody, 1 out of 29 (3.4%) patient had anti‐HMGCR antibody, 1 out of 29 (3.4%) patient had anti‐Jo‐1 antibody. Positive antinuclear antibody was observed in 27 out of 28 (96.4%) patients. Seven out of 23 (30.4%) patients had anti‐SSA antibody, and none of patients had anti‐SSB antibody.

After diagnosis of IIMs with AMAs, all patients but three received corticosteroids. Twenty‐six patients had additional immunotherapies including methotrexate (*n* = 22), cyclophosphamide (*n* = 2), tacrolimus (*n* = 3), rituximab (*n* = 2), intravenous immunoglobulin (*n* = 11), and plasma‐exchanges (*n* = 1).

### Muscle involvement

Limb weakness was observed in 28 out of 37 (75.7%) IIMs patients with AMAs. All 28 patients exhibited proximal limb weakness. Among them, 16 (43.2%) had weakness in both upper and lower limbs, 17 (45.9%) had upper limb weakness, and 27 (73.0%) had lower limb weakness. Additionally, 21 (56.8%) patients displayed neck weakness, while 7 (18.9%) experienced dysphagia. Dyspnea was present in 14 (37.8%) patients, with 4 of them exhibiting Type II respiratory failure. Pulmonary function tests revealed mild‐to‐severe restrictive ventilatory impairment in 6 out of 7 (85.7%) patients. Diaphragm thinning and reduced mobility were detected in 4 out of 8 (50.0%) cases. Myalgia was reported by 10 (27.0%) patients, and muscle atrophy was observed in 2 (5.4%) patients. The median MMT8 total score was 74.5 (IQR 66–78). Cardiac involvement was noted in 19 (51.4%) patients, and among the 9 patients who underwent cardiac magnetic resonance imaging, late gadolinium‐enhanced images in the left ventricular wall were observed in 5 out of 9 (55.6%) patients.

### Pathological features

Muscle biopsy was performed in all 37 IIMs patients with AMAs. Prior to the biopsy, 10 patients had received a short course of oral prednisone therapy (<1 mg/kg/day). Muscle pathological results were classified as follows: IMNM in 30 (81.1%) cases, PM in 2 (5.4%) cases, and nonspecific myositis in 5 (13.5%) cases. None of the pathological results were classified as DM (Table 2).

**Table 2:** Histopathological findings of IIMs patients with AMAs.

Histopathological findings	Patients (*n* = 37)
Myofiber pathology	
Microinfarction, *n* (%)	3 (8.1%)
COX‐deficient/decreasing myofibers, *n* (%)	12/36 (33.3%)
Ragged red fibers, *n* (%)	2 (5.4%)
Succinate dehydrogenase positive fibers, *n* (%)	6/36 (16.7%)
Vasculopathy	
NSE hyperchromatic capillaries, *n* (%)	18 (48.6%)
Immune‐related changes	
MHC‐I expression, *n* (%)	37 (100.0%)
Diffuse MHC‐I in myofiber membrane, *n* (%)	10 (27.0%)
MHC‐I enhancement in perifascicular regions, *n* (%)	2 (5.4%)
Sarcolemmal MAC deposition, *n* (%)	26/36 (72.2%)
Capillary MAC deposition, *n* (%)	7/36 (19.4%)
Connective tissue	
Regional perimysial edema, *n* (%)	7 (18.9%)
Fibrosis perimysial, *n* (%)	5 (13.5%)
Fibrosis endomysial, *n* (%)	4 (10.8%)
Inflammation domain	
Granulomatous inflammation, *n* (%)	1 (2.7%)
CD3+ infiltration, *n* (%)	18 (48.6%)
CD20+ infiltration, *n* (%)	3 (8.1%)
CD68+ infiltration, *n* (%)	32 (86.5%)
Pathological pattern	
IMNM, *n* (%)	30 (81.1%)
PM, *n* (%)	2 (5.4%)
Nonspecific myositis, *n* (%)	5 (13.5%)

AMAs, anti‐mitochondrial antibodies; COX, cytochrome c oxidase; IIMs, idiopathic inflammatory myopathies; IMNM, immune‐mediated necrotizing myopathy; MAC, membrane attack complex; MHC‐I, major histocompatibility complex class I; NSE, nonspecific esterase; PM, polymyositis.

**Figure 1:**
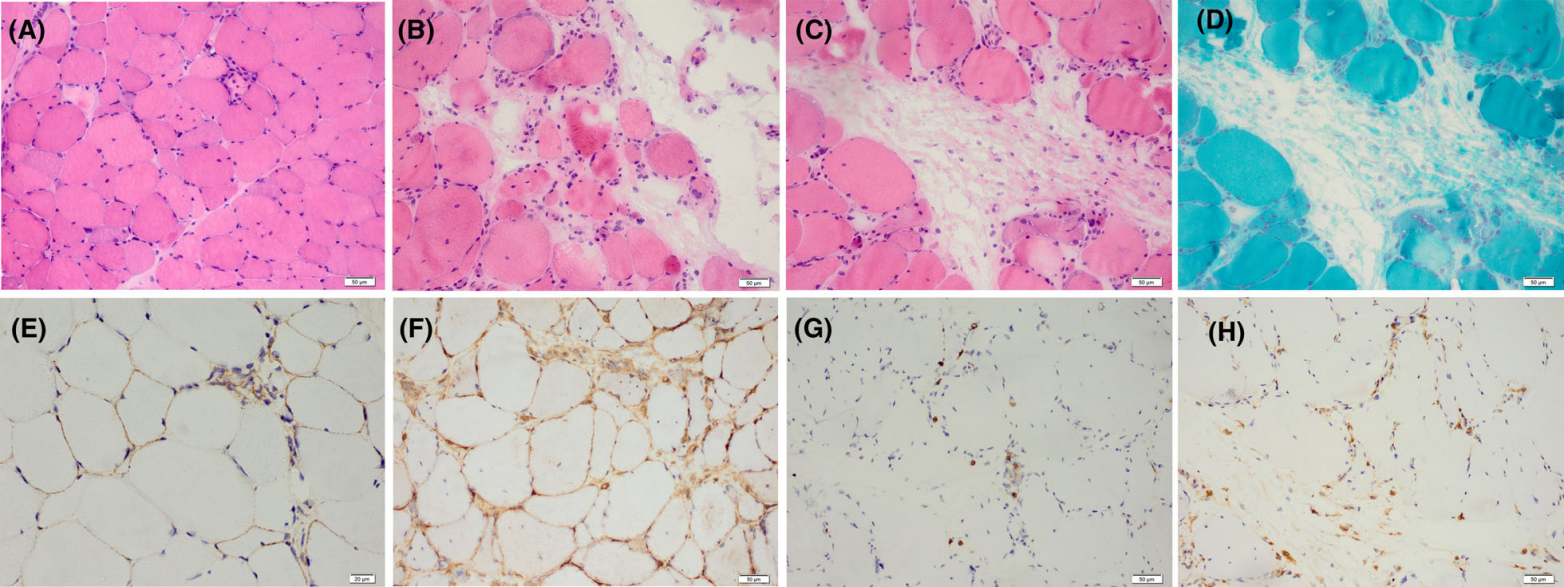
Muscle pathology in AMA‐associated IIMs. (A–C) (H&E staining): scattered necrosis (A), microinfarction (B), regional perimysial oedema (C); (D) (MGT staining): regional perimysial edema. (E) (Immunohistochemistry staining of MAC): deposition of MAC on the sarcolemma of non‐necrotic myofibers. (F) (Immunohistochemistry staining of MHC‐I): diffuse expression of MHC‐I on sarcolemma. (G) (Immunohistochemistry staining of CD8): the endomysial infiltration of CD8+ T cell. (H) (Immunohistochemistry staining of CD68): the perimysial and endomysical infiltration of CD68+ cell. AMAs, anti‐mitochondrial antibodies; IIMs, idiopathic inflammatory myopathies; H&E, hematoxylin and eosin; MGT, modified Gomori trichrome; MAC, membrane attack complex; MHC‐I, major histocompatibility complex class I. Bar: 50 μm in A–D, F, G, and H; 20 μm in E.

Atrophic myofibers and scattered necrosis/regeneration were observed in all patients. Microinfarction was observed in 3 (8.1%) patients. Cytochrome c oxidase‐deficient or decreasing myofibers were found in 12 out of 36 (33.3%) patients. Ragged red fibers were observed in 2 (5.4%) patients, and succinate dehydrogenase‐positive fibers appeared in 6 out of 36 (16.7%) patients. Hyperchromatic capillaries, as observed under nonspecific esterase stain, occurred in 18 (48.6%) patients. All patients exhibited upregulated MHC‐I, with diffuse MHC‐I staining observed in the myofiber membrane in 10 (27.0%) patients and MHC‐I enhancement in perifascicular regions in 2 (5.4%) patients. MAC deposition on the sarcolemma of non‐necrotic myofibers was found in 26 out of 36 (72.2%) patients, while MAC deposition on capillaries was observed in 7 out of 36 (19.4%) patients. Regional perimysial edema was seen in 7 (18.9%) patients, and perimysial and endomysial fibrosis were observed in 5 (13.5%) patients and 4 (10.8%) patients, respectively. Granulomatous inflammation was found in 1 (2.7%) patient. CD3+ cell infiltration was present in 18 (48.6%) patients, with 3 cases showing perivascular infiltration, 5 cases with perimysial infiltration, and 16 cases with endomysial infiltration. CD20+ cell infiltration was observed in 3 (8.1%) patients, with 1 case showing perivascular infiltration and 2 cases with endomysial infiltration. CD68+ cell infiltration was found in 32 (86.5%) patients, with 10 cases showing perivascular infiltration, 17 cases with perimysial infiltration, and 31 cases with endomysial infiltration. Representative histopathological images are shown in Figure 1.

### Serum ccf‐mtDNA and ccf‐nDNA levels

**Figure 2:**
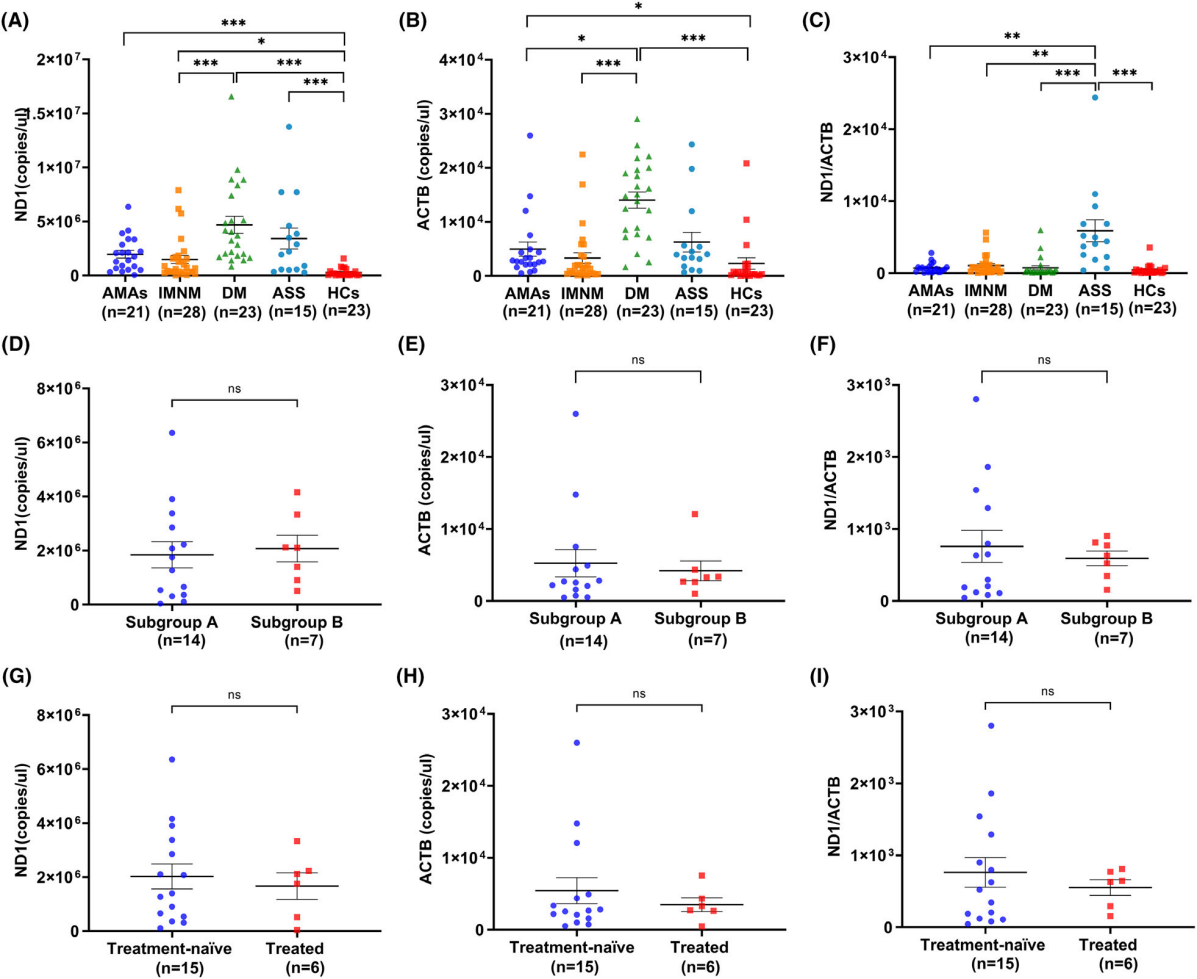
Circulating cell‐free mitochondrial DNA and circulating cell‐free nuclear DNA copy numbers in the sera from AMA‐associated IIMs (*n* = 21), IMNM (*n* = 28), DM (*n* = 23), ASS (*n* = 15), and HCs (*n* = 23). (A) Circulating cell‐free mitochondrial DNA copy numbers in the sera from AMA‐associated IIMs, IMNM, DM, ASS, and HCs. (B) Circulating cell‐free nuclear copy numbers in the sera from AMA‐associated IIMs, IMNM, DM, ASS, and HCs. (C) The ratio of mitochondrial to nuclear DNA in the sera from AMA‐associated IIMs, IMNM, DM, ASS, and HCs. AMAs, anti‐mitochondrial antibodies; IIMs, idiopathic inflammatory myopathies, IMNM, immune‐mediated necrotizing myopathy; DM, dermatomyositis; ASS, anti‐synthetase syndrome; HCs, healthy controls; ND1, circulating cell‐free mitochondrial DNA copy numbers; ACTB, circulating cell‐free nuclear copy numbers; ND/ACTB, the ratio of mitochondrial to nuclear DNA; subgroup A, patients without mitochondrial abnormalities pathologically; subgroup B, patients with mitochondrial abnormalities pathologically. *p < 0.05; **p < 0.01; ***p < 0.001.

We measured absolute serum ccf‐mtDNA (MT‐ND1) and ccf‐nDNA (ACTB) copy numbers in IIMs patients with AMAs, IMNM patients, DM patients, ASS patients, and healthy controls. There were significant differences in the ccf‐mtDNA levels, ccf‐nDNA levels and the ratio of mitochondrial to nuclear DNA among the five groups. Specifically, the serum ccf‐mtDNA levels in IIMs patients with AMAs were significantly higher compared to those in healthy controls (*p* < 0.001), but there were no significant differences between IIMs patients with AMAs and IMNM patients, DM patients, or ASS patients (Fig. 2A). The serum ccf‐nDNA levels in IIMs patients with AMAs were significantly higher compared to those in healthy controls (*p* = 0.02), and significantly lower compared to those in DM patients (*p* = 0.02) (Fig. 2B). There were no significant differences in the serum ccf‐nDNA levels between IIMs patients with AMAs and IMNM patients or ASS patients. The ratio of mitochondrial to nuclear DNA in the sera from IIMs patients with AMAs was significantly lower compared to ASS patients (*p* = 0.002), but there were no significant differences between IIMs patients with AMAs and IMNM patients, DM patients or healthy controls (Fig. 2C).

We subsequently divided IIMs patients with AMAs into patients with mitochondrial abnormalities pathologically and patients without mitochondrial abnormalities pathologically, and compared the ccf‐mtDNA levels, ccf‐nDNA levels and the ratio of mitochondrial to nuclear DNA between them. The ccf‐mtDNA levels in patients with mitochondrial impairment pathologically were higher compared to patients without mitochondrial impairment pathologically, but did not reach statistical significance (Fig. 2D). And there were also no significant differences in the ccf‐nDNA levels and the ratio of mitochondrial to nuclear DNA between the two subgroups (Fig. 2E,F).

In order to determine the possible effect of treatment on the ccf‐mtDNA levels, ccf‐nDNA levels and the ratio of mitochondrial to nuclear DNA, we also divided IIMs patients with AMAs into treated patients and treatment‐naïve patients. The results showed that the ccf‐mtDNA levels, ccf‐nDNA levels and the ratio of mitochondrial to nuclear DNA in treated patients were lower than treatment‐naïve patients, but did not reach statistical significance (Fig. 2G–I).

### Clinical significance of ccf‐mtDNA and ccf‐nDNA levels

We subsequently analyzed the correlation between the clinical profiles (e.g., disease severity and disease activity), pathological markers and the elevated ccf‐mtDNA and ccf‐nDNA levels.

**Figure 3:**
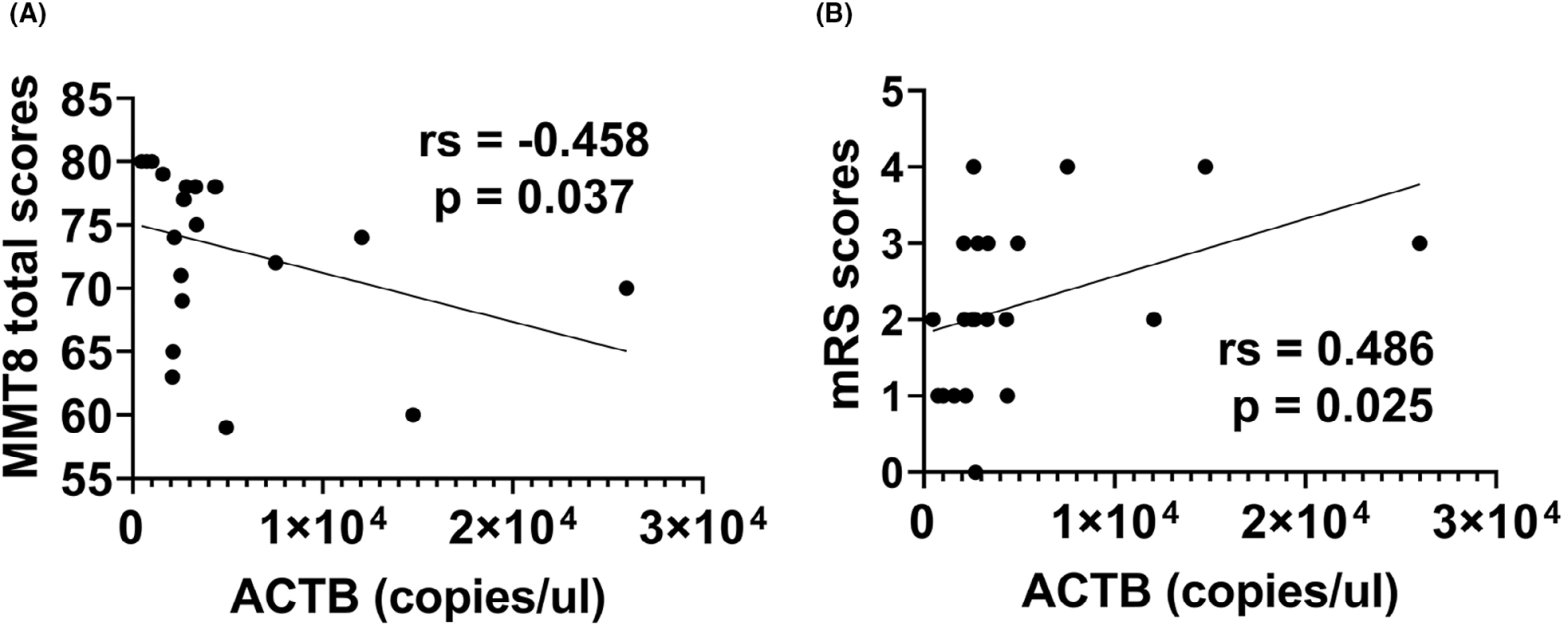
Correlation analysis between MMT8 total scores (A), mRS scores (B), and circulating cell‐free nuclear DNA copy numbers. MMT8, Manual Muscle Testing‐8; mRS, modified Rankin Scale; ACTB, circulating cell‐free nuclear DNA copy numbers.

No significant correlation was found between ccf‐mtDNA levels, ccf‐nDNA levels, and age at disease onset or disease duration (Table S2). However, ccf‐nDNA levels showed a negative correlation with MMT8 total scores (*r*s = −0.458, *p* = 0.037) and a positive correlation with mRS scores (*r*s = 0.486, *p* = 0.025) (Fig. 3). There was no observed correlation between ccf‐mtDNA levels and MMT8 total scores or mRS scores. Additionally, there was no correlation between ccf‐mtDNA levels or ccf‐nDNA levels and the MDAAT scores. Furthermore, ccf‐mtDNA levels and ccf‐nDNA levels did not correlate with the proportion of necrotic myofibers and sarcolemmal MAC deposition.

**Figure 4:**
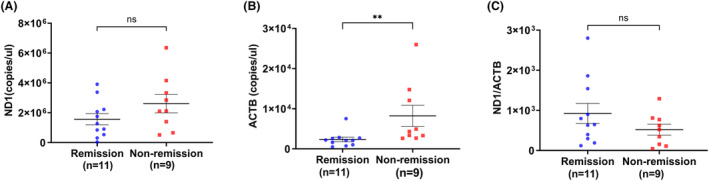
Comparison of ccf‐mtDNA (A), ccf‐nDNA (B) copy numbers and the ratio of mitochondrial to nuclear DNA (C) between remission group (*n* = 11) and non‐remission group (*n* = 9). (A) There were no significant differences in the ccf‐mtDNA copy numbers between remission group and non‐remission group. (B) Ccf‐nDNA copy numbers of non‐remission group were significantly higher than that of remission group. (C) There were no significant differences in the ratio of mitochondrial to nuclear DNA between remission group and non‐remission group. Ccf‐mtDNA, circulating cell‐free mitochondrial DNA; ccf‐nDNA, circulating cell‐free nuclear DNA; ND1, circulating cell‐free mitochondrial DNA copy numbers; ACTB, circulating cell‐free nuclear copy numbers; ND/ACTB, the ratio of mitochondrial to nuclear DNA. **p < 0.01.

Subsequently, we conducted an analysis to determine if the quantification of ccf‐mtDNA and ccf‐nDNA could serve as prognostic markers. Among the 20 patients who had follow‐up visits (median time of follow‐up: 10.0 months; IQR: 4.3–25.3 months), 11 patients achieved remission at their last visit. The non‐remission group exhibited significantly elevated ccf‐nDNA copy numbers compared to the remission group (*p* < 0.01) (Fig. 4A). However, there was no significant difference observed in ccf‐mtDNA copy numbers or the ratio of ccf‐mtDNA to ccf‐nDNA between the two groups (Fig. 4B,C).

## Discussion

The conclusions drawn from current case reports and small‐sample size studies on AMA‐associated IIMs remain inconclusive. There are conflicting findings among different studies. For instance, Hou et al. reported that AMAs occurred only in adult IIMs patients,^9^ while Sabbagh et al. recently reported cases in juveniles.^8^ AMAs were commonly associated with cardiomyopathy.^6^, ^8^ However, some IIMs patients with AMAs presented with skeletal muscle involvement only, without cardiac manifestations.^9^ Based on our cohort, we suggest that AMA‐associated IIMs represent a subgroup of IIMs with specific clinical and muscle histopathological features, primarily affecting adults. In our cohort, the only pediatric patient showed severe Type II respiratory failure and mild limb weakness without cardiac involvement, which is consistent with a previous study.^8^ Therefore, we speculate that pediatric patients may exhibit distinct phenotypes compared to adults and potentially experience more severe symptoms.

Cardiac symptoms initially appeared in 32.4% of the patients, while in 51.4%, they developed over time. The frequency of cardiac involvement also varied, ranging from 16% to 72.4% in different studies.^6^, ^7^, ^8^, ^9^, ^29^, ^30^ Therefore, close attention should be given to the emergence and progression of heart disease throughout the entire duration of IIMs patients with AMAs.

Although limb weakness was the most common initial symptom in our cohort, IIMs patients with AMAs did not have a higher prevalence of limb weakness at disease onset but did exhibit a higher prevalence of limb weakness throughout the disease course (37.8% vs. 75.7%).^8^ The prevalence of limb weakness in IIMs patients with AMAs has been reported to range from 14.3% to 81.8% in different studies.^30^, ^31^ Limb weakness is more frequent than cardiac involvement in our cohort, indicating that limb muscle weakness warrants equal attention as cardiac involvement. Serum CK levels in our cohort of IIMs patients with AMAs were lower compared to those in IMNM patients of our previous study.^32^, ^33^, ^34^ Furthermore, MMT8 total scores in our IIMs patients with AMAs were higher than those in IMNM patients.^35^, ^36^ These findings indicate that limb weakness, although common, tends to be less severe in IIMs patients with AMAs. Uenaka et al. also reported milder limb weakness in IIMs patients with AMAs compared to those without AMAs, suggesting that the presence of AMAs is associated with less involvement of limb muscles.^31^ Another notable clinical feature in our cohort was the prominent involvement of axial muscles, which can be presented as neck weakness, dysphagia, restrictive ventilatory impairment and reduced diaphragm mobility. Neck weakness was found in 56.8% of our patients, similar to that reported in previous studies (57.1–85.7%).^7^, ^9^, ^29^, ^37^ Dysphagia was observed in 18.9% of our patients, and the frequency of dysphagia reported in previous studies ranged largely from 11.8% to 71.4%.^7^, ^8^, ^9^, ^29^, ^30^ Restrictive ventilatory impairment was found in 6 out of 7 (85.7%) patients in our study, which was slightly higher than that in previous studies (31.6–70.6%).^29^, ^37^ Few studies reported diaphragm thinning and reduced mobility in IIMs patients with AMAs, and our findings suggested that reduced diaphragm mobility were found in 4 out of 8 (50%) patients. Trunk weakness was reported in 82.4% of patients in a previous study.^37^ In addition, lordotic posture and dropped head were also reported in IIMs patients with AMAs.^29^, ^37^ Histopathological findings suggested that inflammation appears to be more intense in the trunk muscles rather than in the limbs of IIMs patients with AMAs.^31^ AMAs are regarded as the serological hall markers of PBC, and the frequency of PBC in IIMs patients with AMAs ranged from 14.3% to 75%.^6^, ^7^, ^8^, ^9^, ^29^, ^30^, ^31^ Therefore, IIMs patients with AMAs concomitant with PBC can be classified into overlap myositis.

The coexistence of MAAs and MSAs is common in IIMs. For instance, the anti‐Ro52 antibody is frequently found together with anti‐melanoma differentiation‐associated gene 5 and anti‐Jo‐1 antibodies.^38^ In our cohort, 24.1% of IIMs patients with AMAs also had coexisting MSAs. The frequency of MSAs varied widely from 4.2% to 36.4% in different studies.^6^, ^9^, ^29^, ^30^ Consistent with a previous research,^6^ IIMs patients with the coexistence of AMAs and MSAs likely exhibit pathological characteristics that correspond to the specific MSAs present. Previous studies have shown that skeletal muscle pathology in IIMs patients with AMAs can manifest as DM, PM, IMNM, nonspecific or granulomatous myositis, or even normal/minimal lesions.^6^, ^7^, ^9^ Notably, microinfarction, feature described in regional ischemic immune myopathy,^39^, ^40^ was first observed in our patients. Anti‐NXP2 antibody‐positive DM have been generally associated with microinfarction, indicating vascular damage.^40^ AMA‐associated IIMs may share the same pathogenesis with anti‐NXP2 antibody‐positive DM. However, changes in vascular morphology were not prominent in our patients having microinfarction on muscle biopsy, indicating potential vascular dysfunction. In our cohort, no cases of DM were observed, and IMNM was the predominant histopathological finding regardless of the presence or absence of MSAs. The frequency of IMNM in our study (81.1%) was higher than that reported in previous studies (28.6–68.8%).^6^, ^7^, ^8^, ^9^ Given that the majority of IIMs patients with AMAs were classified as IMNM based on myopathological findings, it is advisable to test for AMAs in MSA‐negative patients with IMNM.

Our findings provide compelling evidence that ccf‐mtDNA and ccf‐nDNA levels are significantly elevated in IIMs patients with AMAs compared to healthy controls. Our findings also revealed the serum ccf‐mtDNA levels, serum ccf‐nDNA levels and the ratio of mitochondrial to nuclear DNA in different subtypes of IIMs. Ccf‐mtDNA is successfully used as a biomarker for conditions associated with mitochondrial dysfunction or stress.^41^, ^42^ Ccf‐mtDNA levels are elevated in IIMs patients with AMAs, IMNM patients, DM patients, and ASS patients, and there is no significance between IIMs patients with AMAs and IMNM patients, DM patients, or ASS patients. And mitochondrial alterations in skeletal muscle biopsies from IIMs patients with AMAs ranged from 28.6% to 52.0%,^7^, ^8^ similar to the findings reported in other subtypes of IIMs.^43^, ^44^, ^45^ We guess that mitochondrial dysfunction partially contributes to the pathogenesis of AMA‐associated IIMs, although mitochondrial damage exists but not unique in AMA‐associated IIMs. Ccf‐nDNA is primarily released into the bloodstream from various cellular processes, including NETosis, apoptosis, and necrosis.^13^, ^46^, ^47^ Previous studies have reported elevated levels of cell‐free DNA and abnormal formation of neutrophil extracellular traps in patients with DM/PM.^22^, ^23^ Therefore, we propose that aberrant formation of neutrophil extracellular traps may also contribute to the pathogenesis of AMA‐associated IIMs. Changes of ccf‐mtDNA, ccf‐nDNA levels and the ratio of mitochondrial to nuclear DNA in AMA‐associated IIMs and IMNM are similar, indicating that they may share the same pathogenesis. Further research is warranted to explore mechanisms of IIM‐associated AMAs.

Previous research has suggested that cell‐free DNA could also serve as a valuable biomarker for monitoring disease activity and severity in other conditions, such as systemic lupus erythematosus and rheumatoid arthritis.^48^, ^49^ Our results demonstrate that ccf‐nDNA levels exhibit a negative correlation with the MMT8 total score and a positive correlation with mRS scores, suggesting ccf‐nDNA may serve as a potential marker for disease severity. However, no significant correlation was found between ccf‐mtDNA and the clinical and pathological indicators in our study. And there was also no significant difference in the ccf‐mtDNA levels between patients with mitochondrial abnormalities pathologically and patients without mitochondrial abnormalities pathologically. This may imply that mitochondrial damage is not parallel to tissue damage, and mitochondrial dysfunction partially contributes to the pathogenesis of AMAassociated IIMs. Furthermore, our findings suggest that higher baseline levels of ccf‐nDNA may indicate a poorer prognosis. Ccf‐nDNA might offer better predictive value for clinical trajectories since its presence, unlike ccf‐mtDNA, is directly linked to cell death and the release of cellular contents.^50^ High levels of ccf‐nDNA have been associated with adverse outcomes in various cancers.^17^, ^51^ Pilot studies have shown a strong correlation between elevated ccf‐nDNA levels and adverse events following pediatric heart transplantation mortality and infection treatment.^52^ Scott et al. have proposed that ccf‐nDNA not only serves as a biomarker for assessing the severity of cellular injury but also holds therapeutic potential as a target.^53^

This study had several limitations that need to be acknowledged. First, the prevalence of AMAs in IIMs was relatively low, and the sample size in the present study was small. Second, the sera samples of 6 IIMs patients with AMAs were collected for measurement of the ccf‐mtDNA and ccf‐nDNA levels when they were already undergoing hormone therapy, which could potentially influence the levels of ccf‐mtDNA and ccf‐nDNA. Additionally, the use of serum samples is considered suboptimal compared to the recommended use of plasma samples. Therefore, validating the clinical value of plasma cell‐free DNA could strengthen our findings and further support its potential as a minimally invasive tool for assessing disease severity and predicting prognosis in AMA‐associated IIMs. Finally, our study was a retrospective study, which restricted our further research on the possible effect of treatment on the presence or absence of remission.

## Conclusion

AMA‐associated IIMs exhibit distinct clinical and histopathological features. In cases of IIMs with notable cardiac or axial muscle involvement and MSA‐negative IMNM, testing for AMAs is recommended. Serum ccf‐nDNA is a promising marker for assessing disease severity and predicting prognosis in AMA‐associated IIMs.

## Funding Information

This work was supported by the Beijing Municipal Science and Technology Commission (grant number Z191100006619034).

## Author Contributions

Conception and design: Wei Zhang and Yikang Wang. Data analysis: Yawen Zhao. Interpretation of results: Meng Yu, Luhua Wei, and Yawen Zhao. Writing—original draft: Yikang Wang. Writing—review and editing: Yun Yuan, Zhaoxia Wang, and Wei Zhang.

## Conflict of Interest

The authors declare that they have no conflict of interest.

## Supporting information


Table S1

Table S2
