# Supplementary material for: Clinicopathological and circulating cell‐free DNA profile in myositis associated with anti‐mitochondrial antibody
Source: Ann Clin Transl Neurol. 2023 Sep 18;10(11):2127–38. doi: 10.1002/acn3.51901 (PMC10647000; doi:10.1002/acn3.51901)
Supplement: Supplementary file 1 — Table S1 [file ACN3-10-2127-s002.docx]

Table S1 Demographic characteristics of IMNM patients, DM patients, ASS patients and HCs

|  | IMNM  (n=28) | DM  (n=23) | ASS  (n=15) | HCs  (n=23) |
| --- | --- | --- | --- | --- |
| Female, n (%) | 22 (78.6%) | 16 (69.6%) | 12 (80.0%) | 18 (78.3%) |
| Age, median (IQR), years | 53 (48, 60) | 60 (52, 64) | 52 (34, 59) | 48 (45, 59) |

IMNM, immune-mediated necrotizing myopathy; DM, dermatomyositis; ASS, anti-synthetase syndrome; HCs, healthy controls. IQR, interquartile range.
