# Supplementary material for: Clinicopathological and circulating cell‐free DNA profile in myositis associated with anti‐mitochondrial antibody
Source: Ann Clin Transl Neurol. 2023 Sep 18;10(11):2127–38. doi: 10.1002/acn3.51901 (PMC10647000; doi:10.1002/acn3.51901)
Supplement: Supplementary file 2 — Table S2 [file ACN3-10-2127-s001.docx]

Table S2 Correlation analysis between ccf-mtDNA, ccf-nDNA copy numbers and clinical, pathological indicators

|  |  | ccf-mtDNA | ccf-nDNA |
| --- | --- | --- | --- |
| Clinical indicators |  |  |  |
| Age at disease onset | rs | -0.083 | 0.252 |
|  | p | 0.721 | 0.270 |
| Disease duration | rs | 0.046 | -0.084 |
|  | p | 0.844 | 0.717 |
| MMT8 total scores | rs | -0.424 | -0.458 |
|  | p | 0.055 | 0.037* |
| mRS scores | rs | 0.220 | 0.4862 |
|  | p | 0.337 | 0.025* |
| MDAAT scores | rs | 0.136 | 0.285 |
|  | p | 0.556 | 0.211 |
| Pathological scores |  |  |  |
| The proportion of necrotic myofibers | rs | -0.210 | 0.305 |
|  | p | 0.403 | 0.218 |
| The proportion of sarcolemmal MAC  deposition | rs | -0.141 | -0.208 |
|  | p | 0.590 | 0.422 |

Ccf-mtDNA, circulating cell-free mitochondrial DNA; ccf-nDNA, circulating cell-free nuclear DNA; MMT8, Manual Muscle Testing-8; mRS, Modified Rankin Scale; MDAAT, Myositis Disease Activity Assessment Tool; MAC, membrane attack complex. *, p < 0.05.
